# Enhancing Interfacial Strength of Epoxy Adhesive Joints Using Chemically Surface-Modified Palm Fibers: Influence of Fiber Loading, Surface Chemistry and Orientation

**DOI:** 10.3390/polym18101180

**Published:** 2026-05-12

**Authors:** Iclal Avinc Akpinar, Simay Bayramoglu, Salih Akpinar

**Affiliations:** 1 Office of Occupational Health and Safety, Erzurum Technical University, 25050 Erzurum, Turkey; 2 STM Defense Technologies Engineering and Trade Inc., 06530 Ankara, Turkey; simay.bayramoglu54@erzurum.edu.tr; 3 Department of Mechanical Engineering, Erzurum Technical University, 25050 Erzurum, Turkey; salih.akpinar@erzurum.edu.tr

**Keywords:** natural fiber, nitric acid oxidation, surface functionalization, epoxy interface, adhesion, polymer

## Abstract

In materials science, the increasing use of lightweight and multi-material structures has made improving the interfacial bonding characteristics of polymer-based adhesive systems increasingly important. Accordingly, chemical surface activation and interfacial engineering strategies have attracted considerable attention for enhancing polymer–fiber compatibility and adhesion performance. However, the combined effects of fiber content, surface treatment, and orientation on adhesion behavior remain insufficiently understood. In the present study, natural fibers obtained from the rachis part of the palm tree were chemically modified and incorporated into an epoxy adhesive matrix to investigate the effect of surface functionalization on polymer–fiber interfacial adhesion. In the first stage, the effects of fiber ratios (5–20 wt%) and chemical surface treatments (methanol cleaning and methanol +2–6% HNO_3_) on adhesion behavior were evaluated. Tensile tests showed that specimens treated with methanol cleaning followed by 4% HNO_3_ oxidation and containing 10 wt% fiber exhibited an approximately 48% increase in failure load compared to neat joints. In the second stage, the influence of fiber orientation (0–90°) was examined using the optimized parameters. The results indicate that interfacial load-transfer capability increased as the fiber orientation approached perpendicular alignment, reaching maximum performance at 90°. Based on SEM observations, nitric acid treatment was found to increase the surface roughness of the fibers and strengthen the polymer–fiber interfacial bond. FTIR, XPS and contact angle measurements suggested the development of oxygen-containing surface functionalities and improved wettability, consistent with enhanced interfacial adhesion. These findings demonstrate that appropriate chemical surface treatment, fiber content, and orientation can effectively enhance the interfacial adhesion and bonding efficiency of epoxy-based adhesive systems, providing practical guidance for the design of high-performance bonded structures.

## 1. Introduction

Adhesive bonding methods are widely used in fields such as automotive, aerospace, defense, marine, and energy systems due to their suitability for joining complex material combinations. As an alternative to traditional joining techniques, this method provides more uniform load distribution [1], offers geometric design flexibility and eliminates negative effects such as tearing, drilling, or cross-sectional weakening in the material. Consequently, improving the performance of adhesively bonded joints has become a significant research focus in modern materials engineering. Among the studies conducted to improve the performance of adhesively bonded joints, methods such as roughening the adherend surface using mechanical or chemical techniques, obtaining appropriate surface energy, optimizing curing conditions, and improving joint geometry stand out. However, one of the most remarkable of these applications is increasing the joint strength by adding various nano-scale additives into the adhesive.

The literature contains numerous examples where the integration of different nanomaterials, such as carbon nanotubes, graphene, and metal oxides (Al_2_O_3_, TiO_2_), has successfully improved joint characteristics [2,3,4]. In a study conducted by Çakır and colleagues [5], the impact of adding various ratios of graphene nanoparticles (GNPs) to epoxy adhesive on the shear and bending behavior of joints formed by bonding glass fiber reinforced polymer (GFRP) plates was investigated. Graphene nanoparticles were incorporated into the epoxy adhesive at weight fractions of 0.1%, 0.2%, 0.3%, 0.4%, and 0.5%. While the shear strength of the bonded joints with pure epoxy was 4.532 MPa, the addition of 0.3 wt% graphene nanoparticles to the epoxy increased the shear strength of the joint to 11.852 MPa, resulting in an improvement of approximately 145%. Furthermore, it was reported that samples containing 0.2 wt% GNP achieved a 100% increase in flexural strength. In a study conducted by Karimi et al. [6], the impact of integrating nanoparticles (fullerenes and single-walled carbon nanotubes) into the adhesive on the static strength and fatigue life of single-lap and hybrid (adhesive and mechanical joining) joints was investigated under various environmental conditions.

Based on the analysis of the study’s findings, it is evident that joints reinforced with nanoparticles exhibit improved performance in terms of both static load capacity and fatigue resistance under various environmental scenarios (such as hygrothermal conditions). Furthermore, it was emphasized that nanoparticles provide a positive contribution for maintaining joint performance under different environmental influences. In a study conducted by Çakır and colleagues [7], the mechanical performance of single-lap joints developed by adding nanostructures improved with halloysite nanotubes (HNT), hexagonal boron nitride (h-BN), and their hybrids into the adhesive was examined. The addition of 2.5 wt% h-BN to the adhesive enhances the shear strength by 57.24% and the flexural strength by 43.98% compared to the neat sample. Furthermore, the hierarchical physical arrangement between HNTs and h-BN flakes creates an effective three-dimensional reinforcement network that promotes crack deflection and energy dissipation through geometric complementarity, leading to an extended crack path and increased energy consumption. As observed in previous research, it was observed that these nanostructure additives improve critical parameters such as the load-carrying capacity, energy absorption, and crack propagation resistance of the joint. However, the difficulty of achieving a homogeneous distribution of nanostructures, high processing costs, and limitations in preventing crack growth remain significant factors that restrict the widespread use of these materials.

Consequently, the incorporation of fibrous structures into the adhesive is considered an alternative strategy aimed at arresting crack propagation within adhesively bonded joints. Thirunavukarasu et al. [8] conducted a study to examine the mechanical performance of adhesively bonded single-lap composite joints reinforced with unidirectional and bidirectional glass fibers and bidirectional carbon fibers under tensile, shear, bending, and free-vibration loading conditions. The tensile strength, flexural strength, and stiffness of the joints increased by 23.9%, 31%, and 19.6%, respectively, when the adhesive layer was impregnated with carbon fiber fabric instead of being unreinforced. In another study, Akpinar et al. [9] experimentally and numerically examined the mechanical behavior of single-lap joints bonded with a composite adhesive incorporating carbon and glass fiber fabrics. Single-lap joints were manufactured using the cured DP460 structural adhesive, with AA2024-T3 aluminum alloy as the adherend material and carbon fiber and glass fiber fabrics as the reinforcing layers. The results showed that the incorporation of fiber reinforcements into the adhesive layer effectively hindered crack propagation within the joint, thereby increasing the failure load by approximately 5% to 41%. However, for carbon and glass fibers to be efficiently utilized in adhesive systems, improving the fiber–epoxy interfacial bonding remains a critical step [10,11,12]. In fiber-reinforced epoxy systems, insufficient interfacial adhesion leads to premature fiber pull-out and interfacial debonding, which significantly reduces load transfer efficiency and macroscopic strength. For instance, a statistically significant correlation between interfacial adhesion strength and tensile strength has been reported in carbon-fiber-reinforced epoxy systems, providing clear mechanistic evidence that interfacial properties play a decisive role in governing composite strength [13]. Therefore, chemical surface modifications introduce oxygen-containing functional groups (–OH, –COOH, etc.) on the fiber surface, permitting hydrogen bonding and covalent interactions, which increase surface roughness and considerably enhance interfacial adhesion with the epoxy matrix [14,15]. In addition, another effective approach to improve fiber–resin/epoxy interfacial interactions involves electrochemical oxidation combined with various silane coupling agents to increase the surface activity of the fibers [16,17].

In recent years, there has been a growing shift toward natural fiber reinforcement, which is one of the low-cost, environmentally friendly, easy-to-process, and interface-modifiable reinforcement options. Natural fibers are preferred as reinforcement elements in structural composite systems due to their low density, high specific strength, environmental friendliness, low production costs, and ease of modification using chemical methods. Natural fibers, which yield positive results in composite systems, are also expected to have positive effects in adhesive bonding systems. However, most existing studies have focused on individual parameters such as fiber content or surface treatment, while the combined effects of fiber content, chemical surface modification, and fiber orientation on adhesive joint performance have not been systematically investigated. This gap limits a comprehensive understanding of interfacial load transfer mechanisms and optimization strategies in fiber-reinforced adhesive systems. Therefore, in this study, natural fibers obtained from the rachis section of the palm tree were reinforced with DP190 structural adhesive. In this study, the natural fiber ratio and the chemical surface treatment parameters applied to the fibers were considered, and the mechanical changes occurring in single-lap joints were investigated using tensile tests. Furthermore, the optimum fiber reinforcement and surface treatment combination was determined, and the effects of different fiber stacking angles within the adhesive on bond performance were examined. Along with the analysis of the experimental results, FTIR, XPS, SEM, and contact angle measurements were employed to assess surface chemistry, fiber–epoxy interfacial interactions, and chemical bonding mechanisms. Thus, the potential for improvement that natural fiber reinforcement can provide to adhesive joints in terms of additive amount, chemical modification, and orientation was comprehensively demonstrated.

## 2. Materials and Experimental Procedure

### 2.1. Materials

**Figure 1:**
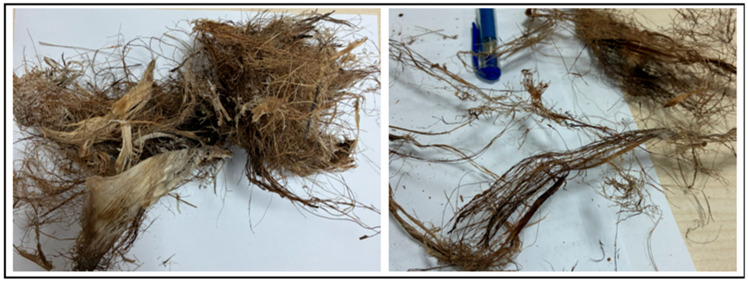
Fiber bundles taken from the rachis section of the date palm tree.

A two-component epoxy-based DP190 adhesive (produced by 3M Company, St. Paul, MN, USA) was used as a structural adhesive to bond the aluminum sheets. The adhesive consists of an epoxy resin and an amine-based curing agent mixed at a 1:1 volume ratio. Detailed chemical parameters are not disclosed by the manufacturer, but to ensure consistency, all samples were prepared under the same conditions. AA2024-T3 aluminum alloy, commonly used in the aerospace and automotive industries, was used as the bonded material. The chemical composition of the alloy used in this study was determined as (wt%): Cu 4.48, Mg 1.57, Mn 0.58, Fe 0.17, Zn 0.16, Si 0.06, Ti 0.03, Others ≤ 0.04, with the balance Al [15]. The relatively high copper content is known to significantly influence surface oxide characteristics and interfacial adhesion behavior, particularly in epoxy-based adhesive systems. As for the fiber structure added to the adhesive, fibers from the rachis section of the palm tree sourced from India, as shown in Figure 1, were used. The mechanical properties of the adhesive, the bonded aluminum material, and the raw fiber are also given in Table 1. For chemical processing of the natural fibers, 99% pure methanol and 57% pure nitric acid (HNO_3_) (these chemicals were sourced from Sigma-Aldrich Chemical Company (MO, USA)) were used.

### 2.2. Natural Fiber Preparation and Surface Treatment

**Figure 2:**
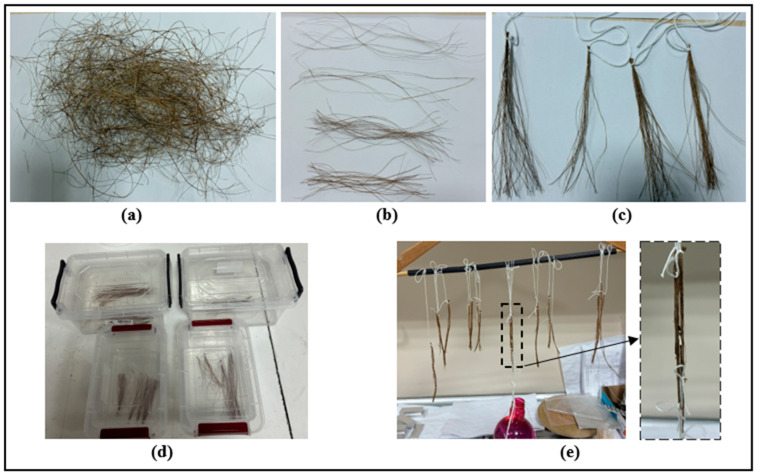
Surface treatments applied to fibers: (**a**) raw fibers with a diameter of approximately 0.30 mm, (**b**) raw fibers of different lengths, (**c**) bundling of fibers of the same length, (**d**) application of chemical surface treatments to fibers, and (**e**) drying processes of fibers.

The raw natural fibers obtained from the palm rachis had diameters ranging from approximately 0.30 to 0.40 mm. To achieve a uniform diameter of approximately 0.25 mm, the fibers were passed through a perforated plate with 0.25 mm holes. The fibers passed through the perforated plate had a diameter of approximately 0.25 mm, and any residues on the surface of the fibers were removed (Figure 2a). As shown in Figure 2b, fibers of different lengths were bundled together so that their lengths were close to each other (Figure 2c). Then, all fibers were first washed in a methanol solution for 1 h. After washing, they were dried by being kept in an oven at 50 °C for 3 h. A portion of the fibers washed with methanol solution were subjected to chemical surface treatment for 1 h in nitric acid solutions of 2%, 4%, and 6% by weight (*w*/*w*), which were prepared by diluting concentrated nitric acid (57 wt%) with distilled water (Figure 2d). After this process, the wet fibers were dried by hanging them for one day under normal ambient conditions, as shown in Figure 2e. The reason for attaching weights to the fibers while hanging them to dry is to ensure that the fibers are straightened during the drying process.

### 2.3. Surface Cleaning Treatments Applied to the Bonded Material (Aluminum Alloy)

**Figure 3:**
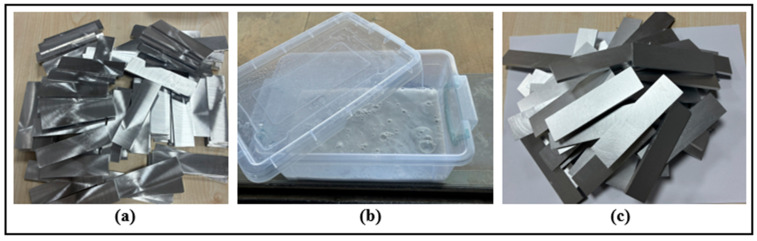
(**a**) Mechanically sanded aluminum surfaces, (**b**) chemical etching with NaOH solution, and (**c**) aluminum surfaces with the oxide layer removed and ready for bonding.

According to the geometry given in Figure 3a, AA2024-T3 aluminum alloy plates with dimensions of 100 × 25 × 4 mm were prepared. To increase the adhesion between the aluminum surface and the adhesive in single-lap bonding joints, a series of mechanical and chemical pretreatments were applied to the surfaces. In the first stage, the aluminum samples were mechanically abraded for approximately 10 min using P800 type SiC sandpaper (purchased from Atlas sandpaper company, Kocaeli, Turkey) to remove any oil, dirt, and rust residues that might be present on the surface. After the abrasion process, the surfaces were thoroughly cleaned with pure water.

Following mechanical cleaning, a chemical etching process was initiated to remove the naturally occurring oxide layer on the aluminum surface. For this purpose, the samples were immersed in a 15% by weight sodium hydroxide (NaOH) solution for 15 min and then rinsed with pure water (Figure 3b). Subsequently, to remove any remaining basic residues and neutralize the surface, the aluminum parts were immersed in a 20% by weight nitric acid (HNO_3_) solution for 3 min and then rinsed again with pure water (Figure 3c). As a result of these processes, a clean and active aluminum surface suitable for bonding was obtained.

### 2.4. Preparation of Fiber Reinforced Adhesives and Joints

**Figure 4:**
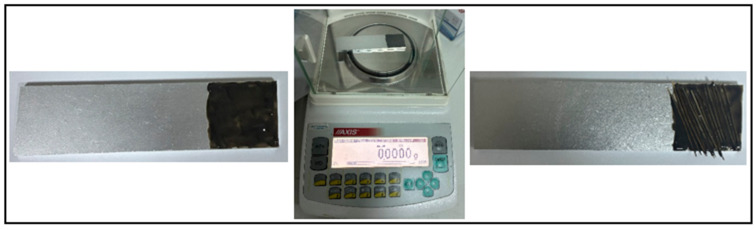
Adhesive and fiber application.

In the presented study, the aluminum plate to be bonded was first weighed using a precision balance. Then, the DP190 adhesive, prepared by mixing epoxy and hardener at a 1:1 ratio, was applied to the surface of the aluminum plate using a gauge and weighed again. Fibers in amounts of 5%, 10%, 15%, and 20% by weight of the adhesive were placed in the adhesive zone at different stacking angles (90, 75, 60, 45, 30, 15, and 0), as shown in Figure 4. The selected parameters were chosen to cover low, intermediate, and relatively high levels within the practically processable range of the adhesive system. Fiber contents of 5, 10, 15, and 20 wt% and HNO_3_ concentrations of 2, 4, and 6 wt% were used to systematically evaluate reinforcement level and surface treatment severity, while fiber orientation angles from 0° to 90° at 15° intervals were selected to examine the effect of alignment relative to the loading direction.

**Figure 5:**
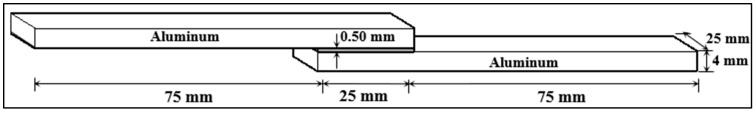
Single-lap joint geometry.

**Figure 6:**
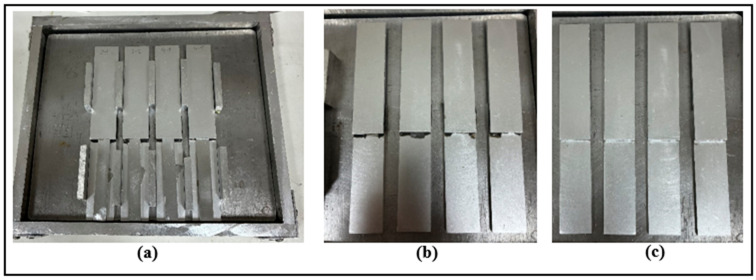
(**a**) Sample production mold, (**b**) some samples after curing, and (**c**) some samples cleaned of residual adhesive.

To produce the single-lap joint with the dimensions given in Figure 5, a specially designed production mold, shown in Figure 6a, was used. Considering the thickness of the fibers impregnated within the adhesive layer, metal pieces of 0.5 mm thickness were placed at the free ends of the specimens to ensure a uniform adhesive layer thickness of 0.5 mm and to improve production repeatability. Additionally, aluminum metal pieces of the same thickness as the samples were placed to adjust the overlap length (Figure 6a). Finally, the DP190 structural adhesive was cured by holding it in a hot press at 70 °C for 120 min, which is the curing condition. After curing, the metal pieces were removed from the joint (Figure 6b) and the excess adhesive that had overflowed during curing was cleaned with a cutting tool (Figure 6c). Sample preparation steps were kept the same for all configurations.

**Figure 7:**
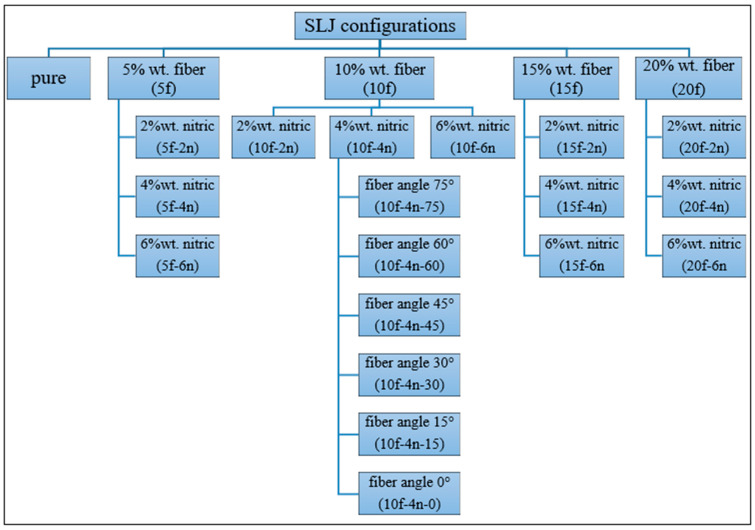
Schematic representation of the categorization of SLJ types.

**Figure 8:**
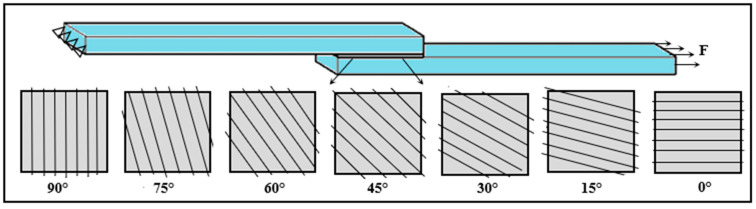
Fiber stacking angles according to the direction of the tensile load.

To facilitate the identification of the tested SLJ configurations, a specimen coding system was established based on the fiber content, surface treatment, and fiber stacking angle (Figure 7). In this notation, F denotes the fiber content (wt%), N denotes the nitric acid treatment concentration (wt%) applied after methanol cleaning, and the final number denotes the fiber stacking angle (°) relative to the loading direction, as illustrated in Figure 8. Accordingly, Pure represents the fiber-free joint; 5F represents the joint containing 5 wt% methanol-cleaned fiber without nitric acid treatment; 5F-2N represents the joint containing 5 wt% fiber treated with methanol cleaning followed by 2 wt% HNO_3_; and 10F-4N-75 represents the joint containing 10 wt% fiber treated with methanol cleaning followed by 4 wt% HNO_3_ and arranged at a fiber stacking angle of 75°. When the stacking angle is not specified in the code, the fiber orientation is 90° relative to the loading direction. Based on this coding system, the SLJ types tested in the study were categorized in Figure 7, while the stacking angle configurations are illustrated in Figure 8. A total of sixty-nine specimens were fabricated, with three specimens produced for each joint configuration shown in Figure 7.

### 2.5. Tensile Test Application

**Figure 9:**
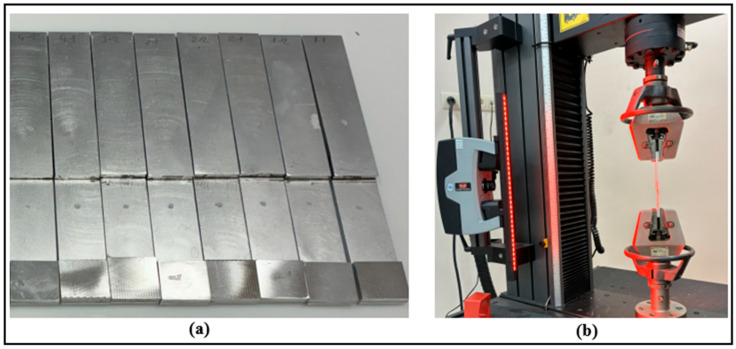
(**a**) Pre-test joint samples; (**b**) tensile test application.

The mechanical behavior of aluminum specimens bonded with adhesive was evaluated using a computer-controlled Instron 5982 (MA, USA) universal testing machine with a 100 kN capacity load cell. The tensile speed was kept constant at 1 mm/min during the experiments. To reduce potential bending effects (eccentricity) that may arise from the load axis in single-lap joints, 25 × 25 × 4 mm aluminum support pieces were placed on both free ends of the specimens prior to testing (Figure 9a).

During the tests, a video extensometer was employed to accurately monitor the displacements in the overlap region (Figure 9b). Accordingly, reference marks were applied on the specimens to define the measurement zone. At the end of the experiments, the failure loads, displacement values, and fracture surface characteristics were obtained for each joint configuration.

After tensile testing, the fracture surfaces of all joints were visually examined and the failure modes were classified according to ISO 10365 [20]. According to this standard, the failure modes in adhesive joints are classified into three main categories: adhesive failure, defined as visible separation at the adhesive–metal interface; cohesive failure, defined as visible fracture within the adhesive layer; and mixed failure, which involves the presence of both adhesive and cohesive failure characteristics. The classification was based on post-test visual inspection of the debonded surfaces and was used to qualitatively compare the dominant failure characteristics of the different joint configurations.

### 2.6. Surface and Chemical Analysis

Fourier Transform Infrared Spectroscopy (FT-IR) analyses were performed to investigate the functional group structure of chemically treated natural fibers and an unreinforced adhesive matrix. For FT-IR measurements, approximately 2 mm^2^ pieces were prepared from each sample and placed in the instrument’s ATR-reflectance unit. Spectra were obtained in the wavelength range of 4000–400 cm^−1^ using a Nicolet iS10 FT-IR/ATR system (Madison, WI, USA). These analyses were carried out to evaluate the reaction-related changes in the surface chemistry of modified and unmodified fibers.

**Figure 10:**
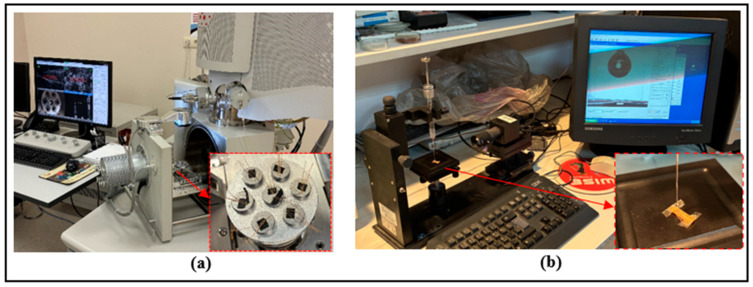
(**a**) Contact angle measuring device; (**b**) Scanning Electron Microscope (SEM) device.

To reveal the differences induced by chemical treatments on the surface morphology of the fibers, imaging was performed using a scanning electron microscope (SEM, FEI Quanta 250, Eindhoven, The Netherlands) (Figure 10a). Thus, microstructural changes affecting the fiber surface roughness, microfibrillation level, and interaction with the matrix were examined in detail.

In addition, static contact angle measurements were performed to determine the wettability properties of surface-treated fibers. Pure water was used as the test liquid in the analyses, and measurements were made with a contact angle measuring device (Dropmeter A-100p, Ningbo Haishumaishi Testing Co., Ltd., Asia-Pacific, China) (Figure 10b). Contact angle measurements were performed using the sessile drop method under ambient laboratory conditions (24 °C and 50% relative humidity). A 5 μL water droplet was deposited from a height of 10 mm, and the image was recorded after 1 s. For each surface condition, five measurements were performed at different locations, and the contact angle values were evaluated using Young–Laplace fitting. The surface free energy of water was taken as γL = 72.8 mJ/m^2^ [21]. The contact angle formed on the solid surface is an indicator of the surface energy and is directly related to the adhesion potential of the surface. The adhesion work (WA), which defines the energy required to separate two phases per unit area, was calculated using Equation (1), as given in the literature [22]. In the equation, θ is the contact angle:

(1) $$W_{A}=\gamma_{L}(1+\cos\theta )$$

To determine the surface chemical composition of untreated, methanol-cleaned, and HNO_3_-treated palm rachis fibers, X-ray photoelectron spectroscopy (XPS, Specs-Flex, Berlin, Germany) was employed. The survey spectra were acquired in the fixed analyser energy mode using a pass energy of 100 eV and an energy step of 1.0 eV. For each treatment condition, FT-IR measurements, XPS analyses, and SEM observations were repeated on independently prepared samples to confirm reproducibility, and the spectra and micrographs presented in the manuscript are representative results.

## 3. Results and Discussion

### 3.1. The Effect of the Natural Fiber Additive Ratio

**Figure 11:**
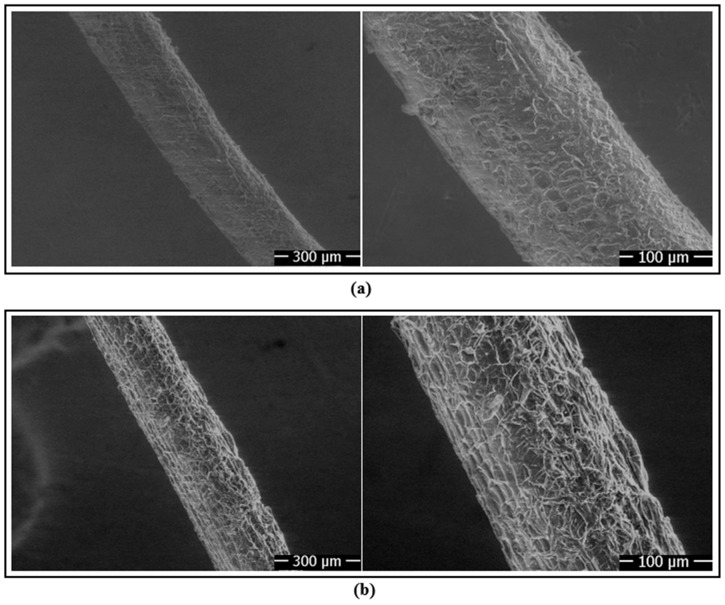
(**a**) SEM image of fibers without surface cleaning treatment; (**b**) SEM image of fibers with surface cleaning treatment.

The mechanical performances of naturally fiber-reinforced and unreinforced single-lap joints (SLJs) subjected to methanol surface cleaning treatment under tensile load were evaluated. SEM images of the fibers before and after surface cleaning treatment are shown in Figure 11. On the untreated fiber surfaces, the presence of tylosis-like particles embedded within the fiber structure, which adversely affect surface smoothness, can be clearly observed (Figure 11a) [23]. After surface cleaning with methanol, it was observed that these particles were significantly removed, and consequently, regular pore structures emerged on the fiber surface (Figure 11b). Furthermore, SEM analyses showed that fiber diameters ranged from 230 to 265 µm and that the chemical cleaning process did not negatively affect the fiber geometry.

**Figure 12:**
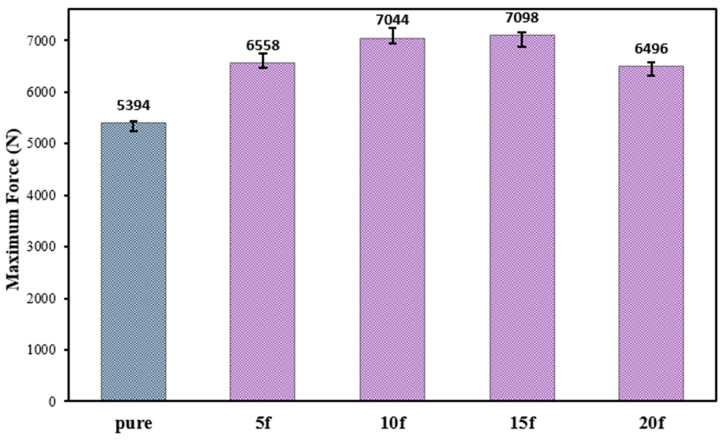
Tensile failure loads of joints reinforced with varying proportions of natural fiber.

When the tensile test results given in Figure 12 are examined, it is seen that the addition of 5% by weight of natural fibers to the adhesive increases the bond failure load by approximately 22%. This improvement can be attributed to the fibers acting as load-bearing elements within the adhesive layer and restricting the propagation of microcracks during loading. It was determined that increasing the fiber additive ratio to 10% and 15% increased the failure load to approximately 31% and 32%, respectively. This indicates that the fiber-adhesive interaction is optimized up to a certain fiber additive ratio. However, increasing the fiber additive ratio to 20% significantly reduces the improvement in bond performance. At higher fiber ratios, the available adhesive becomes insufficient to properly surround and wet the fibers, which can promote local aggregation, interfacial defects, and stress concentrations in the adhesive layer. Consequently, the stress transfer efficiency between the epoxy matrix and the fibers decreases, which facilitates earlier crack formation and limits the expected strengthening effect.

In conclusion, natural fiber reinforcement is an effective approach for enhancing the mechanical performance of adhesively bonded joints, and fiber contents in the range of 10–15 wt% can be considered optimal in terms of load-carrying capacity.

### 3.2. The Effect of Fiber Surface Treatment

**Figure 13:**
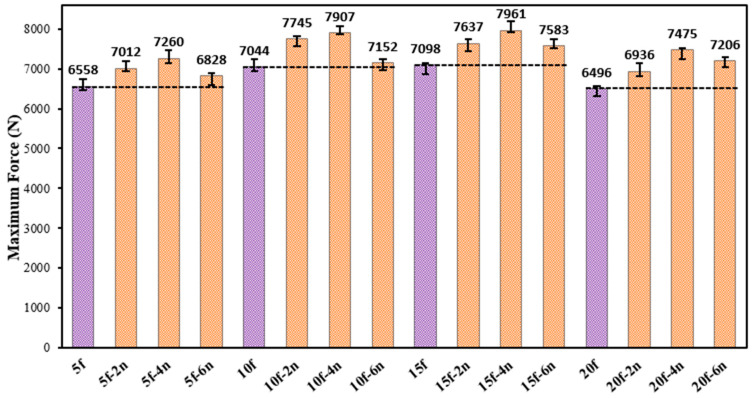
Average tensile failure loads of fiber-reinforced joints subjected to different rates of chemical surface treatment.

To improve the interfacial interaction of the natural fibers added to the adhesive with the epoxy matrix, chemical surface treatment was applied to the fibers using nitric acid (HNO_3_) solutions at concentrations of 2%, 4%, and 6% by weight. The average failure loads of single-lap adhesive joints produced with chemically modified fibers under quasi-static tensile load are given in Figure 13. The results of these failure loads show that chemical surface treatment has a significant and systematic effect on the mechanical performance of natural fiber-reinforced joints.

An analysis of the failure loads presented in Figure 13 indicates that, for joints containing 5 wt% fiber, chemical surface treatments at 2, 4, and 6 wt% increase the average failure load by approximately 4% to 11%. Similarly, for joints reinforced with 10 wt% fiber, the effect of chemical treatment ranges from about 1% to 12%, while for joints with 15 wt% fiber, the improvement reaches approximately 7% to 12%. Notably, in joints containing 20 wt% fiber, the contribution of chemical surface treatment to joint performance increases to the range of 11–15%. This situation demonstrated that chemical surface treatment plays a more critical role in improving interface interaction at high fiber additive ratios.

As mentioned in the previous section, increasing the fiber content from 5% to 20% by weight results in a limited decrease in bond performance in untreated joints due to insufficient wetting at the epoxy–fiber interface. However, when chemical surface treatment is applied to the fibers, this negative effect is significantly reduced and even reversed. This can be attributed to the fact that surface treatment with nitric acid increases the fiber surface energy, resulting in a stronger interface with the epoxy matrix.

**Figure 14:**
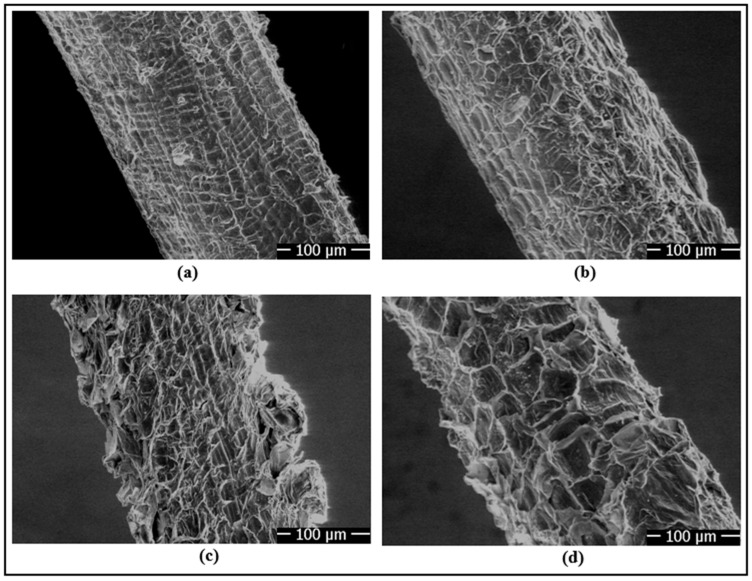
SEM images of chemically surface-treated fibers: (**a**) fiber surface cleaned with methanol, (**b**) fiber chemically surface-treated with 2 wt% HNO_3_, (**c**) fiber chemically surface-treated with 4 wt% HNO_3_, and (**d**) fiber chemically surface-treated with 6 wt% HNO_3_.

Figure 14 shows the SEM analyses performed to investigate the effects of chemical surface treatments on fiber surface morphology. In fibers cleaned only with methanol, a smooth morphology with a limited number of shallow pits was observed on the fiber surface (Figure 14a). However, in fibers treated with 2% nitric acid by weight, more pronounced porous structures and cellular formations appeared on the surface (Figure 14b). With increasing HNO_3_ concentration to 4 wt% and 6 wt%, the density of pores, voids, and micro-irregularities became more evident (Figure 14c,d), indicating progressive surface etching and morphological modification. These observations suggest an increase in the available surface features for mechanical interlocking with the epoxy matrix. However, no direct roughness parameters (e.g., Ra or Rq) were measured in the present study; therefore, the SEM results are interpreted qualitatively. The improved interfacial interaction is further supported by the contact angle/adhesion work results and the mechanical test data.

**Figure 15:**
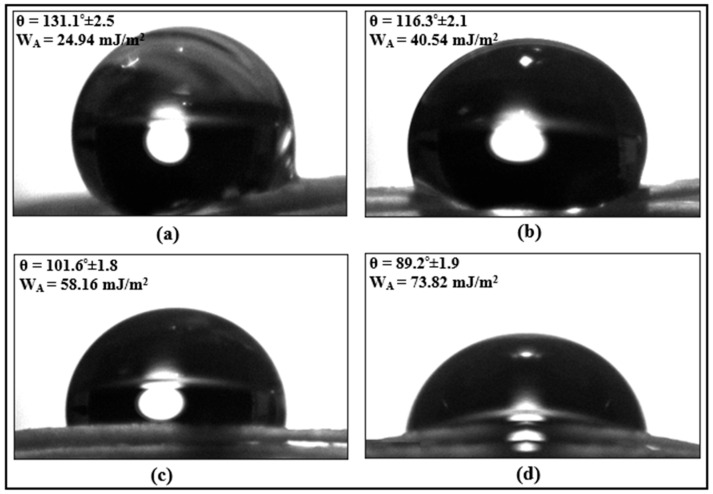
Contact angle measurement images of chemically surface-treated fibers: (**a**) fiber surface cleaned with methanol, (**b**) fiber chemically surface-treated with 2 wt% HNO_3_, (**c**) fiber chemically surface-treated with 4 wt% HNO_3_, and (**d**) fiber chemically surface-treated with 6 wt% HNO_3_.

The effect of chemical surface treatment on the wettability properties of fibers was evaluated by contact angle measurements and adhesion work calculations (Figure 15). The contact angle value of 131.1° obtained from methanol-cleaned fibers indicates that the surface has a distinctly hydrophobic character (Figure 15a). In contrast, in fibers chemically treated with nitric acid, a clear transformation of the surface morphology from a hydrophobic to a hydrophilic structure is observed (Figure 15b–d). Especially when using a 6% by weight nitric acid solution, the significant decrease in the contact angle reveals that the fiber surface acquires an almost completely hydrophilic character. This decrease in contact angle directly contributes to an increase in the adhesion work between the liquid and the fiber surface. The calculated adhesion work values in chemically surface-treated fibers increase by approximately 62% to 196% compared to methanol-cleaned fibers. This increase indicates that the fiber surface energy has increased and the molecular interactions between the epoxy and the fiber have strengthened. Increased adhesion allows for more efficient load transfer between the epoxy and the fibers, directly supporting the improvement in the performance of the adhesive joints. Consequently, it can be said that chemical surface treatment with nitric acid solution significantly improves the fiber surface morphology, wettability, and interfacial bonding characteristics, which contributes significantly to the mechanical performance of adhesive joints, especially at high fiber contents.

### 3.3. The Effect of Fiber Orientation Angles in Natural Fiber-Reinforced Bonding Joints

Based on the quasi-static tensile test results obtained for different fiber additive ratio and nitric acid (HNO_3_) surface treatment concentrations, the optimum parameter combination in terms of joint performance was identified as 10 wt% fiber reinforcement with 4 wt% nitric acid-treated fibers. Accordingly, the effect of fiber orientation angle on joint behavior was investigated using the 10F–4N configuration as the reference. It is well established that the alignment of fibers at different angles relative to the loading direction plays a critical role in determining joint performance.

**Figure 16:**
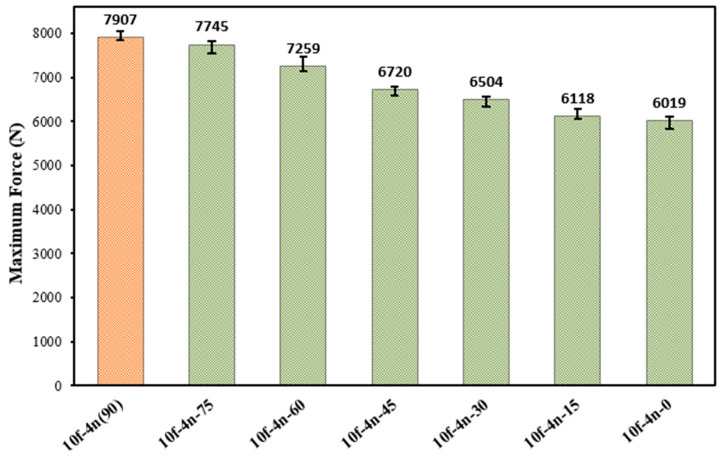
Average tensile failure loads of fiber-reinforced joints with different fiber orientation angles.

An examination of the average failure loads presented in Figure 16 clearly shows a progressive reduction in joint performance as the fiber orientation angle decreases from 90° to 0°. Taking the fiber structure-free connection (pure) as a reference, it was found that the failure load increased by approximately 46% in connections with a 90° fiber orientation (10f-4n (90)) where the fibers are placed perpendicular to the load direction However, it was determined that the increase in failure load in joints with a 0° fiber orientation (10f-4n-0) where the fibers are placed parallel to the load direction was approximately 11%. This situation reveals that fiber orientation is a critical parameter determining joint performance.

Placing the fibers at perpendicular or near-perpendicular angles to the loading direction allows them to act as more effective barriers against crack propagation within the adhesive layer. In such configurations, an advancing crack is forced to follow a longer and more tortuous path around the fiber structure, which increases energy dissipation and delays fracture development. These orientations also improve local stress transfer across the fiber–epoxy interface. In contrast, when the fibers are aligned parallel to the loading direction, their ability to interrupt crack propagation is more limited, resulting in a lower contribution to joint strengthening. Furthermore, it is observed that the increase in bond performance follows an approximately linear trend at intermediate fiber orientation angles between 0° and 90° (15°, 30°, 45°, 60°, and 75°) given in Figure 16. This linear behavior indicates that the contribution of the fibers to load transfer gradually comes into play depending on the orientation angle. In particular, at intermediate-to-high angles such as 60° and 75°, the fibers are believed to contribute simultaneously to load transfer and interfacial strength. In conclusion, these findings demonstrate that in fiber-reinforced adhesive joints, not only the fiber content and chemical surface treatment but also the fiber orientation within the adhesive layer plays a decisive role in governing joint performance.

### 3.4. FT-IR Analysis for Surface-Treated and Untreated Fibers

**Figure 17:**
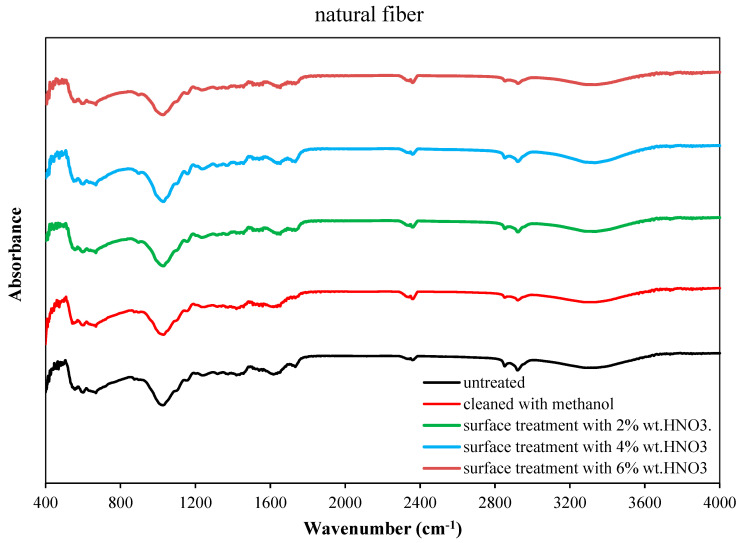
FTIR spectra of untreated and chemical surface-treated natural fibers.

Figure 17 presents the FT-IR spectra of natural fibers obtained from the rachis section of the palm tree in their untreated, methanol-cleaned, and surface-treated states with different concentrations of nitric acid (2%, 4%, and 6% wt. HNO_3_) after methanol cleaning. Examination of all spectra clearly reveals the characteristic functional groups specific to the lignocellulosic structure of the fibers.

In the FTIR spectrum of the untreated fibers, the broad band observed in the range of 3200–3400 cm^−1^ is attributed to the O–H stretching vibrations of hydroxyl groups present in cellulose and hemicellulose. This band indicates the hydrophilic nature of the fibers and their hydrogen bond formation potential. The peaks appearing around 2920–2850 cm^−1^ correspond to the C–H stretching vibrations of aliphatic –CH_2_ and –CH_3_ groups, indicating the presence of waxes, oils, and low-molecular-weight extractives on the fiber surface. The band located at approximately1730–1740 cm^−1^ is associated with the C=O (carbonyl) stretching vibrations of acetyl and ester groups in hemicellulose and is a typical feature of lignocellulosic fibers. The bands in the range of 1600–1510 cm^−1^ are assigned to the aromatic ring vibrations of lignin, while the band at 1240–1260 cm^−1^ represents C–O stretching vibrations originating from lignin and hemicellulose. The strong band observed in the region of 1030–1060 cm^−1^ is related to the C–O–C and C–O stretching vibrations of the cellulose backbone.

In fibers cleaned with methanol, a significant reduction in the intensity of C–H bands, particularly in the 2920–2850 cm^−1^ range, is observed. This indicates that methanol removes wax, oil, and extractive components from the fiber surface, creating a cleaner and more active surface. Simultaneously, the partial reduction in the 1730–1740 cm^−1^ band suggests that ester-containing components on the surface have been removed. This cleaning process allows for better wetting of the fiber surface by the epoxy.

In chemical surface treatments performed with HNO_3_ solution, it is clearly observed that the O-H band, especially in the 3200–3400 cm^−1^ range, is broadened and the intensity of the carbonyl peaks around 1730–1740 cm^−1^ is increased. This suggests that HNO_3_ treatment promotes the surface oxidation of the fibers and increases the relative presence of oxygen-containing functional groups such as –OH, –C=O, and –COOH. Meanwhile, the reduction in the intensity of the bands attributed to lignin in the range of 1600–1510 cm^−1^ suggests that nitric acid partially removes or chemically transforms lignin and hemicellulose components on the fiber surface. As the HNO_3_ concentration increases to 4 wt%, the bands associated with oxygen-containing functional groups become more pronounced, suggesting enhanced oxidative modification of the fiber surface. The cellulose-related band at 1030–1060 cm^−1^ remained observable after treatment; however, because no quantitative peak-ratio analysis was performed in the present study, the degree of oxidation and the preservation of the cellulose backbone were interpreted qualitatively. In contrast, treatment with 6 wt% HNO_3_ showed spectral changes consistent with more severe oxidation, potentially indicating excessive surface etching.

In conclusion, FT-IR analyses show that methanol cleaning activates the fiber surface by removing contaminants, while chemical surface treatments with nitric acid strengthen the fiber–epoxy interface interaction by increasing the amounts of oxygen-containing functional groups on the fiber surface.

### 3.5. X-Ray Photoelectron Spectroscopy Analysis

**Figure 18:**
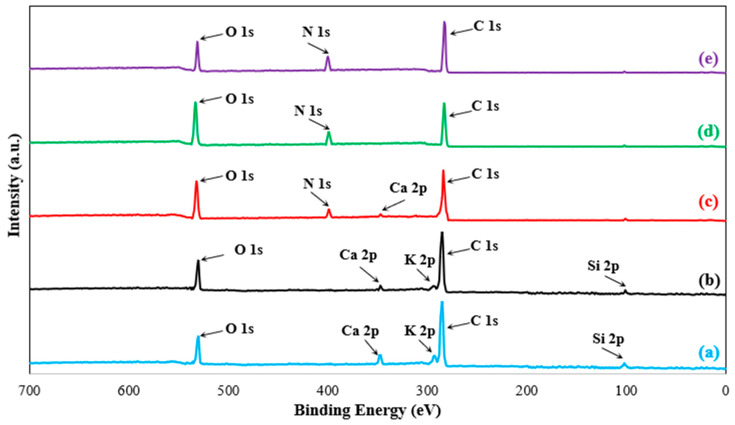
XPS survey spectra of the surfaces of (**a**) (untreated), (**b**) (cleaned with methanol), (**c**) (surface treatment with 2 wt% HNO_3_), (**d**) (surface treatment with 4 wt% HNO_3_) and (**e**) (surface treatment with 6 wt% HNO_3_) samples.

The XPS survey spectra of untreated, methanol-cleaned, and HNO_3_-treated palm rachis fibers are shown in Figure 18, and the corresponding atomic compositions are summarized in Table 2. In all samples, the dominant signals are C 1s and O 1s, confirming that the fiber surfaces are mainly composed of carbon- and oxygen-containing species, as expected for a lignocellulosic material. The intense C 1s peak mainly reflects the carbon-based structure of cellulose, hemicellulose, lignin, and the extractive components on the fiber surface. However, the O1s peak originates from oxygen-containing functional groups such as C–O, C=O, and O–C=O groups found in polysaccharide/lignin structures and oxidized surface types. In addition, weak Ca 2p, K 2p, and Si 2p signals are detected in the untreated fiber, indicating the presence of naturally occurring mineral/ash-related components and surface impurities. After methanol cleaning, the relative carbon concentration decreases from 74.517 to 71.701 at.%, while the oxygen concentration increases from 21.185 to 25.612 at.% and the O/C ratio rises from 0.28 to 0.36. Simultaneously, the Ca, K, and Si contents decrease. This trend indicates that methanol removes surface waxes, oils, and low-molecular-weight extractives, thereby exposing a cleaner lignocellulosic surface enriched in accessible oxygen-containing groups. This interpretation is consistent with the FTIR results, which showed a reduction in the aliphatic C–H bands (2920–2850 cm^−1^) and partial weakening of the carbonyl band around 1730–1740 cm^−1^ after methanol cleaning.

Following nitric acid treatment, the XPS data show a clear and systematic increase in the oxidation level of the fiber surface. The oxygen content increases from 27.987 at.% for the 2 wt% HNO_3_-treated fiber to 31.746 at.% and 33.184 at.% for the 4 wt% and 6 wt% HNO_3_-treated fibers, respectively, while the corresponding O/C ratio increases from 0.40 to 0.48 and 0.51. In parallel, the relative carbon content decreases from 69.638 to 66.361 and 65.273 at.%, indicating progressive conversion of the original carbon-rich surface into a more oxygen-functionalized interface. Chemically, this trend is consistent with oxidation-induced enrichment of hydroxyl, carbonyl, and carboxyl-type species on the fiber surface. At the same time, the continuous decrease in Ca, K, and Si contents suggests that nitric acid treatment not only oxidizes the organic surface but also removes residual inorganic/mineral species. An N 1s signal appears only after HNO_3_ treatment and increases from 0.716 to 1.168 at.% with increasing acid concentration, indicating the presence of nitrogen-containing surface species associated with nitric acid treatment, most likely nitrate-derived or other oxidized nitrogen functionalities at the outermost surface. Because these results are based on survey spectra, the exact chemical states cannot be identified with certainty without high-resolution peak fitting. However, the observed changes in elemental composition provide strong evidence of progressive surface oxidation.

Among the treated fibers, the 6 wt% HNO_3_ sample exhibits the highest oxygen concentration and O/C ratio, indicating the highest degree of surface oxidation. However, when the XPS results are considered together with the mechanical and wettability data of the study, the 4 wt% HNO_3_ treatment appears to provide the most favorable balance between chemical functionalization and interfacial performance. In the mechanical tests, the optimum treatment condition was identified as 4 wt% HNO_3_, whereas the contact angle results showed that increasing nitric acid concentration progressively increased surface polarity and wettability. Therefore, although 6 wt% HNO_3_ produces the most highly oxidized surface, 4 wt% HNO_3_ appears to provide a more effective oxidation level for improving fiber–epoxy interfacial interaction without requiring stronger chemical treatment. In this respect, the XPS results support the FTIR and contact angle analyses and confirm that nitric acid treatment enhances the surface polarity and chemical activity of the palm fibers, thereby promoting stronger adhesion with the epoxy matrix.

These findings can be attributed to the combined effect of stronger fiber–epoxy interfacial bonding and surface morphological modification after chemical treatment. Specifically, oxygen-containing functional groups improve wettability and adhesion, while the porous and irregular surface enhances mechanical interlocking and thus bond strength.

### 3.6. Surface Morphologies of Damaged Joints

**Figure 19:**
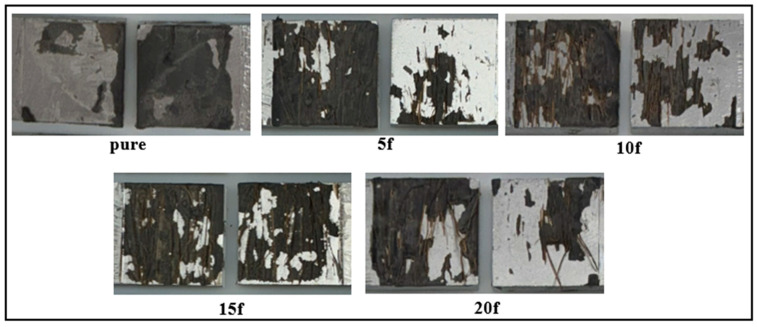
Post-damage surface morphologies of fiber-reinforced joints with varying fiber ratios.

Investigating the damage surfaces of adhesive-bonded joints is critical for understanding the bonding mechanism and load transfer behavior. In this context, the post-damage surface morphologies of fiber structure-free (pure) and fiber-reinforced joints produced with different parameters were evaluated in detail. In the pure joints presented in Figure 19, it can be seen that the adhesion damage mode [20] occurs dominantly. This indicates that the bonding at the adhesive-adherend interface is limited and that under load, cracks propagate directly along this weak interface. The adhesion damage mode is generally associated with low interface energy and insufficient mechanical adhesion. Although no image-based area fraction quantification was performed, the fracture surfaces were systematically classified qualitatively as predominantly adhesive, mixed, or predominantly cohesive in accordance with ISO 10365.

In joints containing natural fiber reinforcement, a pronounced change in fracture surface characteristics is observed (Figure 19). In addition to adhesive failure, cohesive failure within the adhesive layer is also evident in the fiber-reinforced specimens, resulting in a mixed-mode failure mechanism. This behavior indicates that the fibers restrict microcrack propagation within the adhesive matrix, thereby enabling the joints to withstand higher loads before failure.

**Figure 20:**
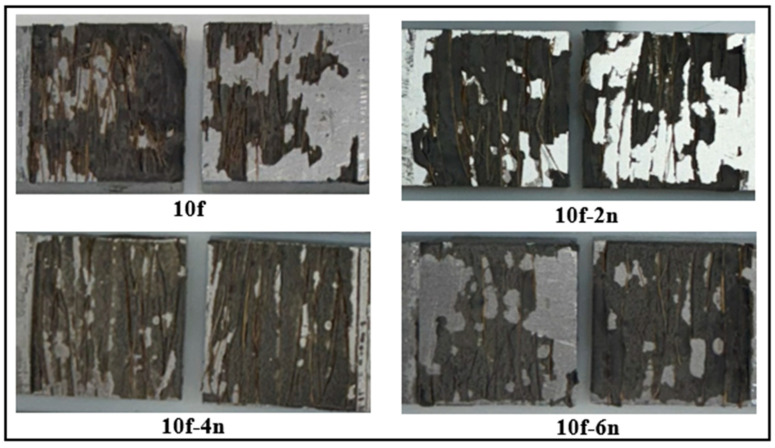
Post-damage surface morphologies of fiber-reinforced joints subjected to chemical surface treatment.

When the damage surfaces of fiber-reinforced joints chemically treated with nitric acid, as shown in Figure 20, are examined, it is clearly seen that the damage mode largely transforms into cohesive damage. This is directly related to the increase in fiber surface energy through chemical treatment and the formation of stronger interfacial bonds between the epoxy matrix and the fiber. Oxygen-containing functional groups (–OH, –COOH) formed on the fiber surface as a result of chemical treatment strengthen the fiber–epoxy interaction by supporting hydrogen bonding and partially covalent bonding mechanisms with the epoxy. Thus, damage occurs within the adhesive rather than along a weak interface, enabling the joint to withstand higher failure loads.

**Figure 21:**
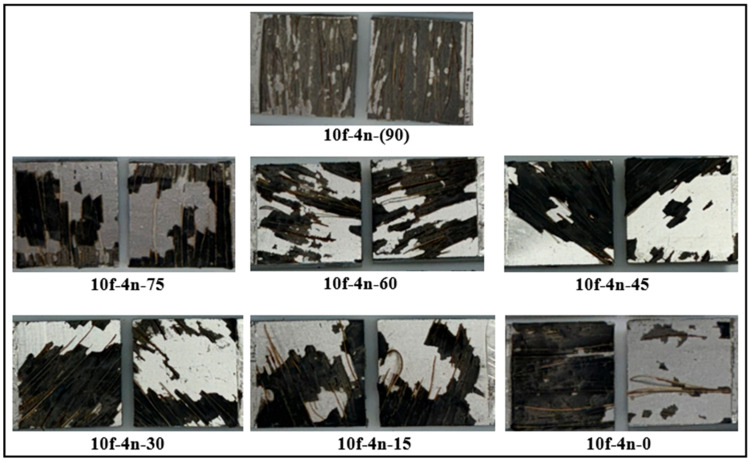
Post-damage surface morphologies of fiber-reinforced joints with different fiber orientations.

The effect of the fiber orientation angle on the damage mechanism is shown in Figure 21. In joints with a 0° fiber orientation, where the fibers are placed parallel to the load direction, it was observed that the damage largely occurred in the adhesion mode. In this case, it was observed that the fibers cannot form an effective barrier against crack propagation and their contribution to load transfer remains limited. In contrast, in fiber orientations between 15° and 75°, a mixed damage mode occurs where adhesion and cohesion damage are observed together. At these angles, the fibers both contribute to load transfer and partially prevent crack propagation, thus delaying the damage process. The most significant improvement was observed in joints with a 90° fiber orientation, where the fibers are placed perpendicular to the load direction. In these samples, the damage occurred almost entirely in the cohesion damage mode. Positioning the fibers perpendicular to the load direction creates an effective mechanical barrier that prevents direct crack propagation, leading to crack elongation and increased energy dissipation. This directly correlates with the test results presented in Figure 16, where joints with a 90° fiber orientation exhibit the highest performance. In contrast, joints with a 0° fiber orientation, where adhesion damage is dominant, show the lowest performance, clearly demonstrating the strong relationship between damage mode and load-carrying capacity.

## 4. Conclusions

This study investigated the mechanical performance of single-lap joints formed by reinforcing natural fibers obtained from the rachis section of the palm tree with epoxy-based structural adhesives. The effects of key parameters such as the fiber additive ratio, chemical surface treatment applied to the fibers, and the fiber stacking angle within the adhesive on the joint performance were experimentally evaluated. Furthermore, the obtained mechanical results were supported by structural and chemical findings using SEM, FT-IR, and contact angle analyses. The main findings of the study are summarized below:

✓ Natural fiber reinforcement was found to significantly increase the load-carrying capacity of adhesive joints. When the fiber additive ratio added to the adhesive was selected between 10% and 15% by weight, effective load transfer within the adhesive was achieved and crack propagation was limited. However, increasing the fiber additive ratio to 20% resulted in only a limited improvement in joint performance due to insufficient wetting and difficulty in achieving homogeneous distribution of the fibers.
✓ It has been shown that chemical surface treatment with nitric acid (HNO_3_) applied to natural fibers significantly strengthens the fiber–epoxy interface interaction. In particular, treatment with 4 wt% HNO_3_ produced a more developed porous/irregular surface morphology and increased surface energy, contributing to improved mechanical interlocking. Under these conditions, the joint performance increased by approximately 48% compared to the unreinforced joints.
✓ SEM images showed that the chemical surface treatment created a porous and rough morphology on the fiber surface, while FT-IR analyses suggested an increase in oxygen-containing surface functionalities (–OH, –C=O, –COOH). Contact angle measurements and calculated adhesion work values confirmed that the fiber surface acquired a hydrophilic character and that the interfacial bonding with the epoxy was strengthened. These microstructural and chemical changes directly support the achieved improvements in mechanical performance.
✓ It was determined that the stacking angle of the fibers in the adhesive plays a significant role in the bonding behavior. In configurations where the fibers were placed perpendicular to the load direction (90°), crack propagation was effectively prevented and the failure load reached its maximum level. However, when the fibers were placed parallel to the load direction (0°), the effect of the fibers on crack propagation remained limited, and the improvement in joint performance was low. At other angles, it was observed that the bonding performance exhibited an approximately linear increase depending on the fiber orientation.
✓ Furthermore, fracture-surface analysis indicated that adhesive failure predominated in unreinforced joints, whereas fiber reinforcement and especially chemical surface treatment shifted the failure behavior toward a more cohesive-dominated mode. This trend is attributed to the strengthening of the fiber–epoxy interface and increased energy absorption within the adhesive layer.

In conclusion, it has been shown that the mechanical performance of natural fiber-reinforced adhesive joints can be significantly improved by using a suitable fiber additive ratio, an effective chemical surface treatment, and an optimum fiber stacking angle together. These findings demonstrate that natural fibers, as a low-cost, environmentally friendly, and sustainable reinforcement option, can serve as a viable alternative to nano and synthetic reinforcements in structural adhesive joints.

## Figures and Tables

**Table 1:** Mechanical properties of the adherend and adhesive used in the study.

	DP 190 [18]	AA2024-T3 [18]	Rachis Fiber [19]
E (MPa)	21 ± 3.2	72400 ± 530	20400 ± 1200
ν	0.35	0.33	-
σ_t_ (MPa)	19.3 ± 1.4	476 ± 17	388 ± 8
ε_t_ (%)	58.2	16	8.2 ± 0.1

E: Young’s modulus; ν: Poisson’s ratio; σ_t_: Ultimate tensile strength; ε_t_: Ultimate tensile strain.

**Table 2:** XPS atomic ratios of chemically treated and untreated natural fibers (%).

Specimen	C	O	N	Ca	K	Si	O/C
untreated	74.517	21.185	-	2.143	1.338	0.817	0.28
cleaned with methanol	71.701	25.612	-	1.527	0.547	0.613	0.36
surface treatment with 2 wt% HNO_3_	69.638	27.987	0.716	1.139	0.151	0.369	0.40
surface treatment with 4 wt% HNO_3_	66.361	31.746	1.046	0.662	-	0.185	0.48
surface treatment with 6 wt% HNO_3_	65.273	33.184	1.168	0.251	-	0.124	0.51

## Data Availability

The original contributions presented in this study are included in the article. Further inquiries can be directed to the corresponding author.
